# Detection of Fraud in a Clinical Trial Using Unsupervised Statistical Monitoring

**DOI:** 10.1007/s43441-021-00341-5

**Published:** 2021-09-29

**Authors:** Sylviane de Viron, Laura Trotta, Helmut Schumacher, Hans-Juergen Lomp, Sebastiaan Höppner, Steve Young, Marc Buyse

**Affiliations:** 1 CluePoints S.A., Avenue Albert Einstein, 2a, 1348 Louvain-la-Neuve, Belgium; 2 Statistical Consultant, Ingelheim Am Rhein, Germany; 3 Boehringer Ingelheim, Biberach an der Riss, Germany; 4 CluePoints Inc., King of Prussia, USA; 5 International Drug Development Institute (IDDI), Louvain-la-Neuve, Belgium; 6 Interuniversity Institute for Biostatistics and Statistical Bioinformatics (I-BioStat), Hasselt University, Hasselt, Belgium

**Keywords:** Statistical monitoring, Central monitoring, Risk-based monitoring, Fraud, Misconduct

## Abstract

**Background:**

A central statistical assessment of the quality of data collected in clinical trials can improve the quality and efficiency of sponsor oversight of clinical investigations.

**Material and Methods:**

The database of a large randomized clinical trial with known fraud was reanalyzed with a view to identifying, using only statistical monitoring techniques, the center where fraud had been confirmed. The analysis was conducted with an unsupervised statistical monitoring software using mixed-effects statistical models. The statistical analyst was unaware of the location, nature, and extent of the fraud.

**Results:**

Five centers were detected as atypical, including the center with known fraud (which was ranked 2). An incremental analysis showed that the center with known fraud could have been detected after only 25% of its data had been reported.

**Conclusion:**

An unsupervised approach to central monitoring, using mixed-effects statistical models, is effective at detecting centers with fraud or other data anomalies in clinical trials.

## Introduction

Risk-based monitoring is a dynamic approach that focuses on oversight activities during the conduct of clinical trials. Applying such monitoring aims to prevent or mitigate risks related to data quality and critical study processes in order to improve patient safety and study outcome due to more reliable data. Findings detected through risk-based monitoring might need additional corrective and preventive actions such as additional training of site staff or clarification of protocol requirements. Additionally, there is a growing consensus that centralized monitoring using statistical techniques is more likely to detect different data anomalies than on-site monitoring or source data verification [1, 2].

A central statistical assessment of the quality of data collected in clinical trials presents “opportunities for new monitoring approaches (e.g., centralized monitoring) that can improve the quality and efficiency of sponsor oversight of clinical investigations.” [1]. Such central statistical monitoring has been suggested to detect fraud and other types of data errors in clinical trials [3–6]. Several examples of data discrepancies suggestive of inappropriate training, poor understanding of the protocol, sloppiness in collecting the data, and fabrication of data or fraud have been reported in the literature [5, 7–10].

In this paper, we use patient-level clinical data from a published trial to show the potential of a statistical assessment of data quality. The trial, known as ESPS2 (Second European Stroke Prevention Study), was conducted in the early 90’s for the prevention of stroke or death in patients with a recent ischemic cerebrovascular event [11]. Among the 60 participating centers, one center randomized more than 400 patients in the trial, most of whom were later found to be actual patients with an ischemic cerebrovascular disease who were never given the investigational therapies [12]. In this paper, we use the database of the trial to show that an unsupervised approach using mixed-effects statistical models might have been effective at detecting the center with fraud earlier in the conduct of the trial.

## Materials and Methods

### The ESPS2 Clinical Trial

The Second European Stroke Prevention Study (ESPS2) was an international multicenter randomized double-blind 2 × 2 factorial design study comparing acetylsalicylic acid (ASA) and/or dipyridamole (DP) to matching placebos in the prevention of stroke or death for patients with a pre-existing ischemic cerebrovascular disease (stroke or transitory ischemic attack within 3 months prior to enrollment). This study randomized a total of 7040 patients in 60 centers across 13 countries and was carried out between February 1989 and March 1995 [11]. A detailed publication on the conduct of this trial reported the fact that serious inconsistencies in case report forms led the trial’s Steering Committee to question the reliability of the data from one center, known as center #2013, which had randomized 438 patients in total. The trial’s Steering Committee made a definitive decision after looking at results from a for-cause analysis of quality control samples of center #2013 that confirmed the implausibility of patient entry. [13].

### Data Quality Assessment (DQA)

Data Quality Assessment (DQA) in clinical trials can use various techniques [11]. In this paper, we use an unsupervised statistical monitoring approach aimed at detecting sites with atypical data patterns compared to all other trial sites. The approach is based on the following principles: (a) data coming from the various centers participating in a trial should be largely similar, save for the random play of chance, and systematic variations that occur in reality (e.g., in multi-regional clinical trials) [5]; (b) a battery of standard statistical tests are applied to the patient data to compare the distribution of the data in one center compared with all other centers [3]; (c) mixed-effects models are used to allow for the natural variability between the centers [7, 9]; (d) tests that are relevant given the type of each variable (continuous, categorical, or date variable) are systematically applied to all patient-level data in a completely unsupervised manner; and (e) an overall “Data Inconsistency Score” (DIS) is computed from the mean, on a log scale, of the *P*-values of all statistical tests performed. For each center, a weighted geometric mean of all *P*-values is calculated with down-weighting of highly correlated tests and a resampling procedure is used to assign a P-value to the geometric mean. [10]. This approach is generally applicable to multicenter trials of sufficient size (in terms of number of centers and volume of data collected) to justify the use of statistical tests. A DIS of 1.3 or larger corresponds to an overall *P*-value less than 0.05 and as such it flags a center whose data significantly differ from the data of all other centers. The larger the DIS, the more discrepant the data are. The operating characteristics of individual statistical tests and the DIS have been studied analytically, through simulations and in real-life examples. The simulations were performed using data from actual trials and contaminating these data over a wide range of parameters corresponding to realistic or extreme situations of data discrepancies [7, 9, 10].

### Detection Strategy

**Fig. 1:**
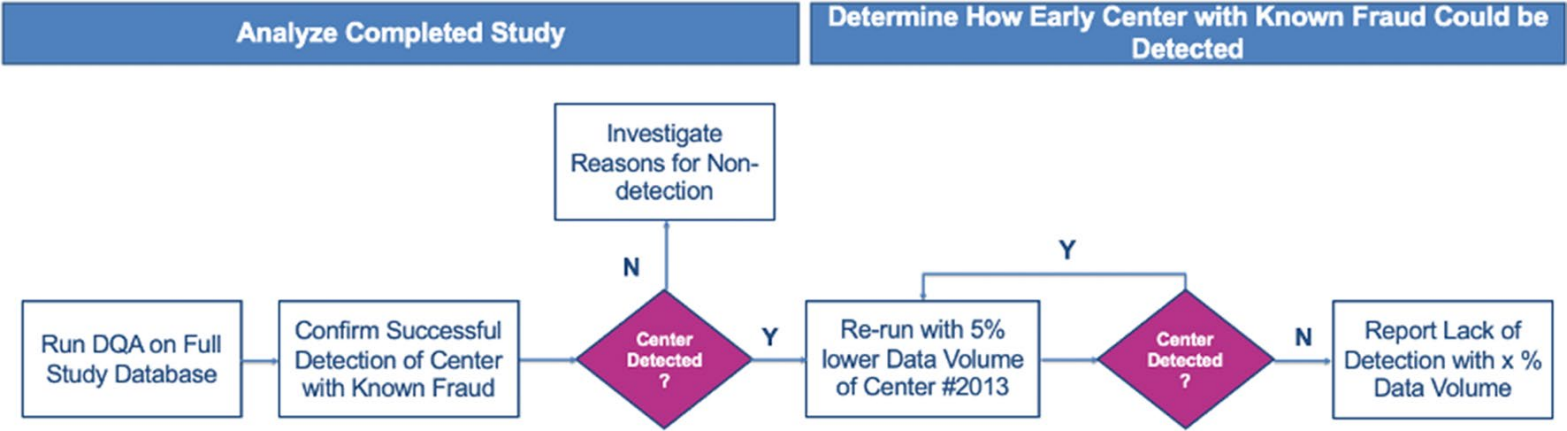
Detection strategy. For DQA re-runs of earlier versions of full study database, the cut-off calendar dates were determined by successive 5% lowering of the data volume for center #2013

The database of ESPS2 trial was transferred for analysis in August 2020. The database contained all clinical data (collected via case report forms) and laboratory results, including unique identifiers for patients, centers, countries, and visits from the 7040 randomized patients. The analyses were conducted in two separate phases (Fig. 1).

The objective of the first phase was to find out whether the DQA could identify the known fraudulent center (center #2013). The DQA team remained blinded to the scope and nature of the known issues until after the analysis was completed and findings presented to the sponsor team. The objective of the second phase was to determine how early issues at the fraudulent center could have been detected. The DQA analysis was iteratively re-executed on versions of the trial database representing progressively earlier timepoints in the progress of the study for center #2013. In particular, the calendar date by which 95% of the patient follow-up visits had been conducted at center #2013 was used as the cut-off date for the first incremental version, and only patient data generated up to this calendar date were included in the analysis. The same process was used to analyze trial database versions representing 90% progress, 85% progress, etc., until center #2013 was no longer detected by the analysis (DIS < 1.3). This approach was used to efficiently detect the minimum cut-off date when center #2013 became atypical.

## Results

### DQA

The DQA analysis was performed on the latest database of the ESPS2 trial. Table 1 shows the number of statistical tests performed by variable domain and category of test. A total of 838 tests were performed for each of the 60 centers in the trial. The “data reporting” category of tests included tests for missing data and reporting rates (count of records by patient and by patient-visit).

**Table 1:** Number of statistical tests performed by variable domain and category of test

Domain	Data reporting^a^	Categorical	Continuous	Count	Date^b^	Correlation	Total
Adverse events	12	21	8	1			42
Clinical events	128	120	48	3	2	2	303
Concomitant medications	1	5					6
Death details	4	2	4	1			11
Disposition	39	33	3				75
Drug accountability	9	9		3			21
Laboratory	13		72				85
Medical history	57	57	24	3			141
Procedures	74	72	8				154
Total	337	319	167	11	2	2	838

^a^Data reporting tests include tests for missing data and reporting rates by patients and visits (count of records per patients or patient-visits)

^b^Date tests look specifically at visits on Saturday/Sunday given that the study did not accept visits and/or assessments being performed on such dates

### Bubble Plot

**Fig. 2:**
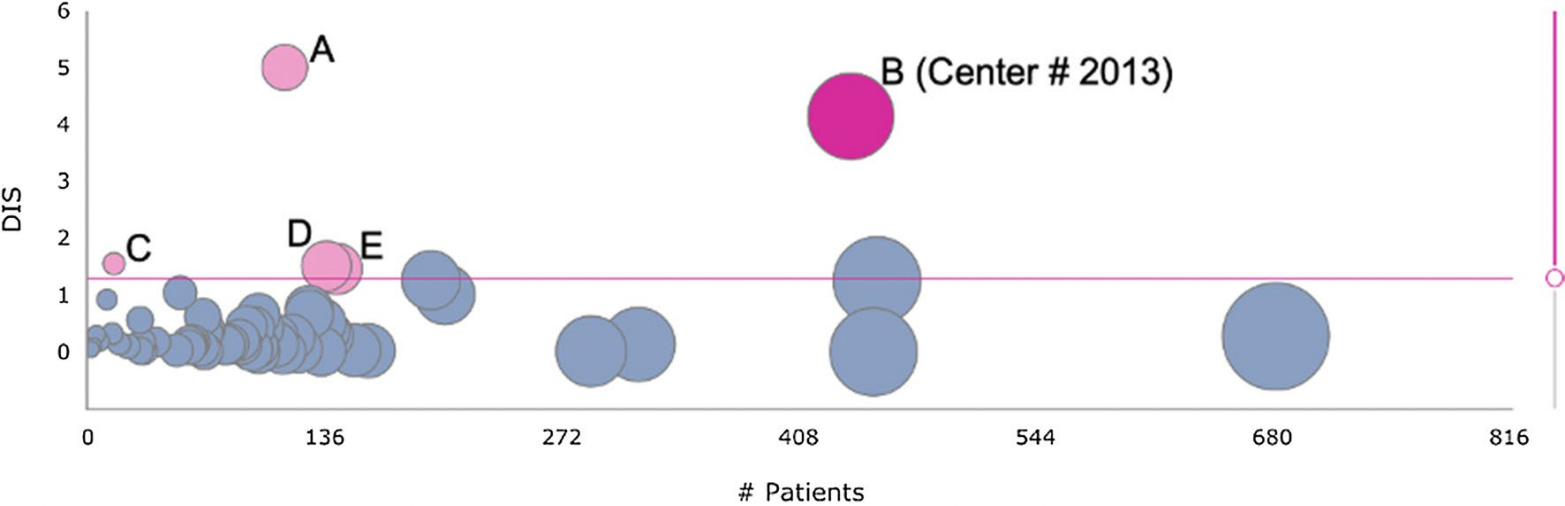
Bubble plot using full study database showing 5 centers with a Data Inconsistency Score (DIS) > 1.3 (*P*-value < 0.05). Center with known fraud is denoted B (Center #2013)

Figure 2 shows the “bubble plot” of the study using the full study database, with each bubble positioned according to the size (x-axis) and DIS (y-axis) of a center. The size of each bubble is proportional to the number of patients randomized in the corresponding center. Of the 60 centers participating in the ESPS2 trial, 5 were identified as atypical (DIS > 1.3, magenta bubbles), including the known fraudulent center, denoted B (Center #2013).

### DQA Findings

Findings for the 5 atypical centers using the full study database are summarized in Table 2. The most atypical center (Center A, DIS = 5.0) exhibited data discrepancies that could all be explained because that center had randomized an atypical patient population. Three atypical centers (Centers C, D, and E) with a lower DIS (1.3 < DIS < 2) would warrant closer scrutiny in an on-going trial. Here we focus on the center with confirmed fraud, which was the second most atypical center in the study (Center #2013, DIS = 4.14) (Table 3).

**Table 2:** Summary of the findings across the 5 most atypical centers in ESPS2 using the full study database. Center #2013 was the center with known ﻿fraud

Center	Number of Patients	DIS (*P*-value)	Primary reasons for center to be outlying
A	113	5 (*P* = 1.10^–5^)	Old population with more diseases and abnormal assessments at baseline as well as more severe bleeding, more abnormal stroke assessments, neurological examinations, and laboratory results at follow-up visits
B (Center # 2013)	438	4.14 (P = 7.10^–5^)	Only SAEs reported (no other AEs reported), low between and within patient variability for laboratory results and vital signs, atypical proportions, and large number of missing values in multiple data domains
C	15	1.55 (*P* = 0.028)	Atypical vital signs and laboratory results (including propagations and outliers), low rate of spontaneous AE complaints, only epistaxis as bleeding event, and high rate of fatal stroke (2 in 15 patients)
D	137	1.51 (*P* = 0.031)	High mean handicap score at baseline, low rate of correct drug usage, high rate of spontaneous AE complaints, more abnormal examinations and laboratory results
E	143	1.46 (*P* = 0.035)	Population with a higher rate of associated conditions and more concomitant medications at baseline as well as more bleedings, more abnormal and atypical laboratory results at follow-up visits, high dropout rate (72 in 143 patents)

*DIS* Data Inconsistency Score

**Table 3:** Comparison of DQA and Sponsor findings for Center #2013

Category	DQA findings	﻿Sponsor findings [14]
Adverse Events	No non-serious AEs reported, too few SAEs, no patients with vascular or bleeding events reported	Very low incidence of adverse events
Vital signs	Very low variability between and within patients at the center for pulse rate and systolic blood pressure	Very low variability in blood pressure readings
Study Drug Compliance	Patients highly compliant with study drug regimen	Very low variability in counts of returned study drug capsules
Clinical Laboratory Data	Very low variability between and within patients at the center for numerous lab measurements	No statistical finding^a^

^**a**^For-cause evaluation of quality control and blood samples for study drug concentrations revealed strikingly different distributions, which were incompatible with prescribed dosages. Subsequent for-cause evaluation of samples for plasma proteins indicated that all blood samples from center #2013 were composed of a mixture taken from a few individuals

### Findings for Center #2013

In Center #2013, only serious adverse events had been reported. Neither non-serious adverse events nor other types of events such as vascular events were reported. There was an atypically low variability between and within the patients of the center for multiple laboratory results and vital signs. Finally, the analysis revealed multiple atypical proportions and missing values in different domains (such as in study drug compliance or adverse events). These findings were consistent with the Sponsor findings, which were previously published [13, 14].

### Incremental DQA Analysis

The incremental DQA analysis was performed on successively smaller data volumes. This approach reproduces what would have happened if the study had been regularly monitored throughout its course. Table 4 shows a few steps of the incremental DQA analysis. Center #2013 could have been detected as being atypical starting at about 25% of the final data volume, with less than half the patients randomized, and about one-third of the number of patient-visits in this center. Of note, centers A and B of Table 2 were detected as being atypical in all incremental analyses with more than 25% of data volume, but centers C, D, and E were not significantly different at all these analyses, while two further centers were marginally significant at some of these analyses (DIS < 2.00).

**Table 4:** Incremental analysis for Center #2013

Data volume	Date	Rank	DIS (*P*-value)	Number (%) patients	Number (%) patient-visits	Number (%) significant tests
100%	20 Jun 1995^**a**^	2	4.14 (*P* = 7.10^–5^)	438 (100%)	3543 (100%)	48 (100%)
75%	7 Dec 1992	2	4.27 (*P* = 5.10^–5^)	391 (89.3%)	2724 (76.9%)	41 (85.4%)
50%	5 Feb 1992	2	2.88 (*P* = 0.001)	284 (64.8%)	1837 (51.8%)	30 (62.5%)
30%	16 Sep 1991	3	1.83 (*P* = 0.015)	241 (55.0%)	1137 (32.1%)	22 (45.8%)
25%	7 May 1991	4	1.73 (*P* = 0.018)	205 (46.8%)	962 (27.2%)	21 (43.8%)
20%	23 Nov 1990	6	1.15 (*P* = 0.07, *NS*)	163 (37.2%)	784 (22.1%)	16 (33.3%)

*DIS* Data Inconsistency Score, *NS* Not Significant

^a^Date of last patient-visit

For comparison with the dates in Table 4, the study team of the ESPS2 trial had suspicions about center #2013 that led to a detailed statistical assessment of the data from this center in June 1992. This assessment led to a for-cause audit in January 1993 and to an expert review of patient compliance to the study treatments through an analysis of blood samples in June 1993, at which time the fraud was confirmed and the center excluded.

## Discussion

Our DQA analyses show that an unsupervised statistical analysis may effectively detect centers that commit fraud. In the ESPS2 trial, the DQA analysis produced overwhelming evidence that the data at center #2013 were inconsistent with the data at all other centers (Data Inconsistency Score = 4.14, P = 7.10^–5^). Incremental DQA analyses showed that the center could have been detected early on in the course of the study. Our analysis is retrospective and based on clean data, which may have facilitated the detection of issues that in reality would have been partly confounded by unclean data.

Had DQA been used in this study on an on-going basis, the centers identified as outliers in Fig. 2 could have been scrutinized with a view to explaining the data discrepancies between these centers and the other centers participating to the trial. As far as Center #2013 is concerned, in-depth inspection of this center, with the findings of the DQA in hand, would have permitted to confront the investigator committing the fraud and to close the site if the responsible investigator could not provide a proper explanation for the data findings. This investigator was in fact convicted after prolonged legal action and extensive additional analyses, including blood concentrations of the investigational drugs, which proved beyond a reasonable doubt that the patients had never received these drugs as mandated by the protocol [12, 15]. All data from Center #2013 were excluded from the final analyses of ESPS2 [14]. Herson [16] discusses the need of implementing “fraud recovery plans” in prospective randomized trials, in case some participating centers are found, based on objective data and before unblinding the trial, that their data are of such dubious quality that they might raise suspicion about the trial findings. In the case of ESPS2, exclusion of center #2013 did not materially affect the results of the trial, which confirms the robustness of the findings obtained in large-scale randomized clinical trials, as long as the fraud affects all treatment groups equally [14]. In such cases, there may be a small dilution of the treatment effect and hence a bias in favor of no difference between the randomized groups, but no bias in favor of the alternative hypothesis.

Other approaches have been proposed for the detection of centers with suspicious data. Van den Bor et al. [17] developed a computationally simple statistical procedure to identify the center with known fraud in the ESPS2 trial, using baseline data only. They, too, succeeded in detecting the center #2013 [17]. We are currently performing a comparison of the types of discrepancies detected by their statistical procedure, when applied to the totality of the data, as compared to the DQA findings reported here.

The true prevalence of fraud in clinical trials is largely unknown [18]. In contrast with other types of experiments, very few publications have been made of fraud cases and no systematic review has been conducted [19]. Cases of confirmed fraud are unfortunately rarely published, which makes research on fraud prevention and detection very challenging. The ESPS2 trial is an exception, with several publications describing the fraud in detail [13, 14]. Sponsors and clinical investigators have an ethical obligation to report fraud, regardless of the consequences, to ensure the transparency and integrity of clinical research and maintain public trust in the process.

Our DQA analyses and the findings of van den Bor et al. [17] confirm that statistical methods are effective at detecting fraud. It was hypothesized more than two decades ago that it is extremely hard for an investigator who commits fraud to invent plausible data [20–22]. This hypothesis has since been confirmed both experimentally [23] and in actual clinical trial datasets [24]. Our simulations indicated that in comparison to dichotomous or categorical variables, continuous variables are more informative for the detection of atypical data. Additionally, the power increased when having more patients and patient-visits. It is advised to use this statistical method in trials having at least 5 sites. Indeed, it has been shown that if there is more than 20% of contamination overall (e.g., similar misunderstanding of protocol requirements or common equipment miscalibration across all centers), the atypical data may not be detected due to lack of sensitivity [10].

Our own experience with central statistical monitoring suggests that overt fraud is rare and limited, although other causes of data errors (including sloppiness and lack of understanding or training) are quite common [3, 5, 7, 8, 10]. For example, data errors, if detected in real time, can be corrected and remedial actions can be taken, including explanations or proper training if required. Finally, the incidence of fraud or data errors may well go down even further if investigators are aware that sophisticated techniques are in place for the automatic detection of atypical data. The cost of these sophisticated techniques pales in comparison with the cost of human data checks and seems justified except in very small trials where a statistical approach would lack power to detect any useful signals.

Increasing the use of DQA in clinical trials might improve patient safety as well as enhance the assessment of efficacy with valid data. Regular monitoring of a trial may not only flag data anomalies and errors, it can also flag trial centers with atypical populations or patients for whom deeper medical review seems indicated. For example, the patient population of center A of the ESPS2 study looked atypical in comparison to the rest of the trial: patients were older and had a higher number of medical histories. Over the course of the trial, they reported more severe bleeding, abnormal stroke assessments or neurological examinations, and atypical laboratory results. Of note, this example also shows that statistical monitoring is indicated for reasons beyond the detection of fraud or misconduct, providing the trial sponsor with a deeper understanding of how the trial is executed at various sites.

## Conclusion

An unsupervised approach to central monitoring, using mixed-effects statistical models, is effective at detecting centers with fraud or other data anomalies in clinical trials. Increasing the use of such methods in the conduct of clinical trials might enhance safety of the patients as well as improve the validity of the outcomes due to more accurate study data.
